# Heme Oxygenase-1 Induction by Carbon Monoxide Releasing Molecule-3 Suppresses Interleukin-1β-Mediated Neuroinflammation

**DOI:** 10.3389/fnmol.2017.00387

**Published:** 2017-11-20

**Authors:** Chih-Chung Lin, Chien-Chung Yang, Li-Der Hsiao, Ssu-Yu Chen, Chuen-Mao Yang

**Affiliations:** ^1^Department of Anesthetics, Chang Gung Memorial Hospital at Linkou, and College of Medicine, Chang Gung University, Tao-Yuan, Taiwan; ^2^Department of Traditional Chinese Medicine, Chang Gung Memorial Hospital at Linkou, Tao-Yuan, Taiwan; ^3^Department of Physiology and Pharmacology and Health Aging Research Center, College of Medicine, Chang Gung University, Tao-Yuan, Taiwan; ^4^Research Center for Industry of Human Ecology, Research Center for Chinese Herbal Medicine, and Graduate Institute of Health Industry Technology, Chang Gung University of Science and Technology, Tao-Yuan, Taiwan

**Keywords:** carbon monoxide releasing molecules, astrocyte, reactive oxygen species, heme oxygenase-1, inflammation, siRNA

## Abstract

Neurodegenerative disorders and brain damage are initiated by excessive production of reactive oxygen species (ROS), which leads to tissue injury, cellular death and inflammation. In cellular anti-oxidant systems, heme oxygenase-1 (HO-1) is an oxidative-sensor protein induced by ROS generation or carbon monoxide (CO) release. CO releasing molecules (CORMs), including CORM-3, exert anti-oxidant and anti-inflammatory effects. However, the molecular mechanisms of CORM-3-induced HO-1 expression and protection against interleukin (IL)-1β-induced inflammatory responses have not been fully elucidated in rat brain astrocytes (RBA-1). To study the regulation of CORM-3-induced HO-1 expression, signaling pathways, promoter activity, mRNA and protein expression were assessed following treatment with pharmacological inhibitors and gene-specific siRNA knockdown. We found that CORM-3 mediated HO-1 induction via transcritional and translational processes. Furthermore, CORM-3-induced HO-1 expression was mediated by phosphorylation of several protein kinases, such as c-Src, Pyk2, protein kinase Cα (PKCα) and p42/p44 mitogen-activated protein kinase (MAPK), which were inhibited by respective pharmacological inhibitors or by gene-specific knockdown with siRNA transfections. Next, we found that CORM-3 sequentially activated the c-Src/Pyk2/PKCα/p42/p44 MAPK pathway, thereby up-regulating mRNA for the activator protein (AP)-1 components c-Jun and c-Fos; these effects were attenuated by an AP-1 inhibitor (Tanshinone IIA; TSIIA) and other relevant inhibitors. Moreover, CORM-3-induced upregulation of HO-1 attenuated the IL-1β-induced cell migration and matrix metallopeptidase-9 mRNA expression in RBA-1 cells. These effects were reversed by an matrix metalloproteinase (MMP)2/9 inhibitor or by transfection with HO-1 siRNA.

## Introduction

Cellular and tissue injuries initiate inflammatory responses to eliminate detrimental factors and injured tissues, thereby facilitating tissue repair. When this process is disturbed, the uncontrolled response produces excessive cell damage that results in tissue dysfunction or chronic inflammation. With brain damage, oxidative stress accompanied by pronounced inflammatory responses accelerates neurodegenerative disorders (Hsieh and Yang, 2013). Several studies of neuronal degeneration indicate that chronic imbalance of redox states and inflammation are major contributors to brain damage (Dröge, 2002; von Bernhardi and Eugenín, 2012). These findings suggest that reactive oxygen species (ROS) are both cell defense molecules and critical regulators of cellular functions including proliferation, differentiation and apoptosis. Previous reports indicate that excessive generation of ROS is associated with tissue injury during brain inflammation and degeneration (Andersen, 2004; Gong et al., 2011). Factors including pro-inflammatory cytokines, microbial infection and peroxidant exposure, promote ROS generation and stimulate expression of several inflammatory genes including inducible nitric oxide synthase (iNOS), cyclooxygenase-2 (COX-2), cytosolic phospholipase A_2_ (cPLA_2_), matrix metalloproteinase-9 (MMP-9) and adhesion molecules, to regulate physiological and pathological processes (Chiurchiù and Maccarrone, 2011; Lee and Yang, 2012; von Bernhardi and Eugenín, 2012). Similarly, our previous study found that interleukin (IL)-1β enhanced nicotinamide adenine dinucleotide phosphate (NADPH) oxidase/ROS signal to induce MMP-9 expression, which initiated rat brain astrocyte (RBA-1) cell migration (Yang et al., 2015a). Therefore, pharmacological interventions have been developed as protective and therapeutic strategies against oxidative stress-induced inflammation and neurodegeneration (Chong et al., 2005; Asaithambi et al., 2011).

Endogenous carbon monoxide (CO) is recognized as a vital anti-oxidant and anti-apoptotic factor, rather than as a toxic molecule (Abraham and Kappas, 2008). Moreover, exogenous CO limits anion superoxide generation by NADPH oxidase (Nox) 4 during inflammation in cerebrovascular diseases and exerts neuroprotective effects in the central nervous system (Basuroy et al., 2009). However, due to the established toxicity in other conditions, there are challenges to using gaseous CO in disease treatment. Therefore, the development of CO-releasing molecules (CORMs) is a major contributor to increasing therapeutic applications. These compounds enable CO delivery to targeted sites without the toxicity of gaseous CO (Romão et al., 2012). CORMs have been extensively investigated and found to up-regulate heme oxygenase-1 (HO-1) expression in various cell types and animal models (Sawle et al., 2005). However, most CORMs are soluble in organic solvents, which restricts their application in clinics. CORM-3, a water soluble compound, is compatible with dissolving in blood and may be useful as therapeutic intervention (Clark et al., 2003; Bani-Hani et al., 2006; Choi et al., 2016). Although several studies support the beneficial effects of CORM-3 in the heart and lungs (Clark et al., 2003; Pak et al., 2016), the detailed mechanisms of signaling molecules involved in HO-1 expression induced by CORM-3 have not been completely elucidated in RBA-1 cells.

In heme metabolism, HO-1 is an oxidative stress-sensitive and anti-oxidant enzyme. HO-1 also possesses cytoprotective properties, regulates redox homeostasis and is anti-inflammatory (Ryter et al., 2006; Paine et al., 2010). Impairment of HO-1-dependent iron metabolism and the presence of oxidative stress interfere with cellular self-protective functions in HO-1-deficient mice, thereby producing liver damage and pro-inflammatory responses (Poss and Tonegawa, 1997). In contrast, overexpression of HO-1 attenuates focal cerebral ischemia-mediated brain damage (Chao et al., 2013). Therefore, HO-1 both catalyzes degradation of pro-inflammatory free heme via enzymatic function and generates several anti-inflammatory compounds such as bilirubin and CO (Abraham and Kappas, 2008). Thus, HO-1 induction promotes anti-oxidant activities, adaptive responses and cell resistance to protect against oxidative injury. Furthermore, several signaling components mediate HO-1 expression in various models. For example, Ryter et al. (2006) found that the non-receptor tyrosine kinases (including c-Src and Pyk2) mediate HO-1 expression via various inducers. Protein kinase Cs (PKCs) are the downstream components of c-Src/Pyk2, and modulate HO-1 induction by acrolein in human bronchial epithelial cells (Zhang and Forman, 2008), by fisetin in human umbilical vein endothelial cells (HUVECs; Lee et al., 2011) and by nerve growth factor (NGF) in PC12 cells (Rojo et al., 2006). In addition, several reports also indicate that mitogen activated protein kinases (MAPKs), including p38, c-Jun N-terminal kinase 1 and 2 (JNK1/2) and extracellular signal-regulated protein kinases 1 and 2 (Erk1/2), activate HO-1 protein expression in response to various stimuli (Elbirt et al., 1998; Kietzmann et al., 2003; Zhang and Forman, 2008; Lee et al., 2011). Furthermore, HO-1 expression activated by protein kinases results from the stimulation of various transcription factors, such as activator protein (AP)-1, by different stimuli in various cell types (Wright et al., 2009; Yang et al., 2012, 2017). However, the relationships between c-Src, Pyk2, PKCα, Erk1/2 and AP-1 activated by CORM-3 and which produce HO-1 expression, remain unknown in RBA-1 cells. The functional effects of HO-1 induction by CORM-3 or adenovirus-introduced HO-1 on IL-1β-mediated responses were also investigated.

The present study aimed to elucidate the signaling pathway underlying CORM-3 effects on HO-1 expression. We found that CORM-3 sequentially activated a c-Src/Pyk2/PKCα/Erk1/2 pathway in RBA-1 cells. The activated signals induced the expression of c-Jun and c-Fos (AP-1 components) and increased HO-1 levels. Furthermore, the functional effect of HO-1 induction by CORM-3 or adenovirus-introduced HO-1 suppressed the IL-1β-induced MMP-9 mRNA expression and cell migration. These effects were reversed by an MMP2/9 inhibitor or by transfection with HO-1 siRNA in RBA-1 cells. The findings have potential implications for developing a novel treatment strategy for preventing neurodegeneration.

## Materials and Methods

### Materials

Dulbecco’s modified eagle’s media (DMEM)/F-12 medium, fetal bovine serum (FBS), Lipofectamine 2000, OPTI-MEM and siRNAs for c-Src (Csk-RSS321555), PKCα (RSS351640), p44 (M61177_Stealth_245), p42 (M64300_Stealth_490), c-Jun (RSS240570) and c-Fos (RSS320772) were purchased from Invitrogen (Carlsbad, CA, USA). Hybond-C membrane and enhanced chemiluminescence (ECL) western blotting detection systems were purchased from GE Healthcare Biosciences (Buckinghamshire, UK). Dimethyl sulfoxide (DMSO), tricarbonylchloro (glycinato) ruthenium (II; CORM-3), enzymes, Pyk2 siRNA (SASI_Rn01_00044067), TRIZOL, the 2,3-bis-(2-methoxy-4-nitro-5-sulfophenyl)-2Htetrazolium-5-carbox-anilide (XTT) assay kit and other chemicals were from Sigma (St. Louis, MO, USA). The BCA protein assay reagent was from Thermo Scientific (Philadelphia, PA, USA). Anti-phospho-c-Src (Tyr^416^, #2101), anti-phospho-Pyk2 (Tyr^402^, #3291), anti-phospho-PKCα/βII (Thr^638/641^, #9375) and anti-phospho-p42/p44 MAPK (Thr^202^/Tyr^204^, #9101) antibodies were from Cell Signaling (Danvers, MA, USA). Anti-phospho-PKCα (Ser^657^, ab180848) was from Abcam (Cambridge, UK), and anti-GAPDH (#MCA-ID4) was from was from Encor (Gainesville, FL, USA). Anti-c-Src (sc-18), anti-Pyk2 (sc-9019), anti-p44 (sc-94), anti-42 (sc-154), anti-PKCα (sc-208), anti-c-Jun (sc-1694) and anti-c-Fos (sc-52) antibodies were from Santa Cruz (Santa Cruz, CA, USA). Anti-HO-1 antibody (ADI-SPA-895), actinomycin D (ActD), cycloheximide (CHI), PP1, SU6656, PF431396, Gö6976, Gö6983, Ro31-8220, U0126 and Tanshinone IIA (TSIIA) were from Enzo Life Sciences (Farmingdale, NY, USA). All primary antibodies were diluted 1:1000 in phosphate-buffered saline (PBS) with 1% bovine serum albumin (BSA). SDS-PAGE reagents were from MDBio Incorporation (Taipei, Taiwan).

### Cell Culture and Treatment

RBA-1 primary culture cells originated from neonatal rat cerebrum astrocytes and naturally developed through successive cell passages (Jou et al., 1985). The cells were cultured in DMEM/F-12 containing 5% FBS, and were made quiescent at confluence by incubation in serum-free DMEM/F-12 for 24 h. Cells were then incubated with CORM-3 at 37°C for the indicated durations. When inhibitors were used, cells were pretreated with the inhibitors for 1 h before exposure to CORM-3. Treatment of RBA-1 cells with DMSO or the pharmacological inhibitors alone had no significant effects on cell viability, as determined by an XTT assay kit (data not shown).

### Protein Preparation and Western Blotting

Cells were washed with ice-cold PBS and harvested in SDS-loading buffer (Tris-HCl, pH 6.8, 0.1 M; SDS, 1%; glycerol, 5%; β-mercaptoethanol, 2.5%; bromophenol blue 0.02%) to yield whole cell extracts. Proteins were separated by SDS-PAGE and transferred by electrophoresis onto Hybond-C membranes. Membranes were incubated with antibodies at 1:1000 in Tween-Tris buffered saline, and an anti-GAPDH antibody was used as an internal control. Membranes were washed with Tween-Tris buffered saline four times for 5 min and then incubated with 1:1500 secondary horseradish peroxidase-conjugated antibody for 1 h. Following washing, immunoreactive bands were detected by ECL and captured using a UVP BioSpectrum 500 Imaging System (Upland, CA, USA). Image densitometry analyses were quantified using UN-SCAN-IT gel software (Orem, UT, USA).

### Total RNA Extraction and Real-Time Polymerase Chain Reaction (RT-PCR) Analysis

Quiescent RBA-1 cells were incubated with 30 μM CORM-3 for the indicated durations, or for 4 h in the presence or absence of the indicated inhibitors. Total RNA was extracted using TRIZOL according to the manufacturer’s protocol, and was then reverse-transcribed to cDNA and analyzed by RT-PCR. RT-PCR was performed using a TaqMan gene expression assay system, with sequences of primers and probes as follows:

- HO-1
  - sense: 5′-TTTCACCTTCCCGAGCAT-3′,
  - antisense: 5′-GCCTCTTCTGTCACCCTGT-3′,
  - probe: 5′-CATGAACACTCTGGAGATGACC-3′
- c-Jun
  - sense: 5′-CCTTGTCCCCCATCGACAT-3′
  - antisense: 5′-CCTTTTCCGGCACTTGGA-3′
  - probe: 5′-CGCATGAGGAACCGCATCGCT-3′
- c-Fos
  - sense: 5′-CCTTGGAGCCGGTCAAGAA-3′
  - antisense: 5′-CGAGCCACTGGGCCTAGAT-3′
  - probe: 5′-ACCCTTTGATGACTTCTTGTTTCCGGCA-3′
- GAPDH
  - sense: 5′-AACTTTGGCATCGTGGAAGG-3′
  - anti-sense:5′-GTGGATGCAGGGATGATGTTC-3′
  - probe: 5′-TGACCACAGTCCATGCCATCACTGC-3′

RT-PCR was performed using a 7500 Real-Time PCR System (Applied Biosystems, Foster City, CA, USA). Relative gene expression was determined using the ΔΔCt method, with Ct indicating threshold cycles. All experiments were performed in triplicate.

### Transient Transfection with Short Interfering RNA (siRNA)

SMARTpool RNA duplexes were obtained from Invitrogen and RBA-1 cells were cultured onto 12-well plates. At 70%–80% confluence, transient transfection occurred with siRNAs (100 nM) corresponding to c-Src, Pyk2, PKCα, p42, p44, c-Jun, c-Fos and scrambled siRNA. GenMute™ reagent was used, followed by mixing with 75 μL GenMute™ transfection buffer. After 10–15 min, 100 μL of the mixture was applied directly to the cells. The cells were washed with PBS and maintained in DMEM/F-12 with 10% FBS for 24 h. Next, cells were starved and changed to serum free media for 48 h. The transfection efficiency (approximate 60%) was determined by transfection with enhanced green fluorescent protein (EGFP).

### Plasmid Construction, Transfection and Luciferase Reporter Gene Assays

A rat HO-1 promoter (accession no. J02722.1; −766 to +20) was constructed (sense primer: GGTACCCAGGAAGTCACAGTGTGGCC; antisense primer: CCCGAGCTCGTCGAGCTGTGGGCGCTCCAT) and cloned into the pGL3-basic vector containing a luciferase reporter system. RBA-1 cells were transfected with plasmid DNA using Lipofectamine 2000. Co-transfection with pCMV-gal encoding for β-galactosidase was used as a control for transfection efficiencies. To assess promoter activity, cells were collected and disrupted by sonication in lysis buffer (25 mM Tris phosphate, pH 7.8, 2 mM ethylenediaminetetraacetic acid, 1% Triton X-100 and 10% glycerol). After centrifugation, aliquots of the supernatants were tested for luciferase activity using a luciferase assay system (Promega, San Luis Obispo, CA, USA). Firefly luciferase activities were standardized to β-galactosidase activity.

### Migration Assay

RBA-1 cells were cultured to confluence in 6-well culture plates and then starved in serum-free DMEM/F-12 media for 24 h. Monolayer cells were manually scratched with a blade to create extended and definite scratches in the center of dishes, with a bright and clear field. Detached cells were removed by washing the cells once in PBS. Serum-free DMEM/F-12 media with or without CORM-3 (30 μM) or IL-1β (3 ng/mL) was added to each well after pretreatment with hydroxyurea (1 μM) and inhibitors for 1 h. Images of migratory cells from the scratch boundary were observed and acquired at 0 and 48 h, using a digital camera and a light microscope (Olympus, Japan). The number of migratory cells was counted from the resulting four phase images from each time point and then averaged across each experimental condition. Four separate assays were conducted to generate the data.

### Statistical Analyses

All data are expressed as means or means ± standard errors of the mean (SEMs) of three individual experiments performed in duplicate or triplicate. Statistical significance was determined for comparisons of western blot data between two groups using paired two-tailed Student’s *t*-tests. Comparisons of multiple groups were conducted using one-way analysis of variance (ANOVA) followed by Tukey’s *post hoc* tests, in GraphPad Prism (GraphPad, San Diego, CA, USA). Statistical significance was indicated by *p* < 0.05. Error bars within the dimensions of the symbols were omitted from the figures.

## Results

### CORM-3 Induces HO-1 Expression and Promoter Activity

**Figure 1:**
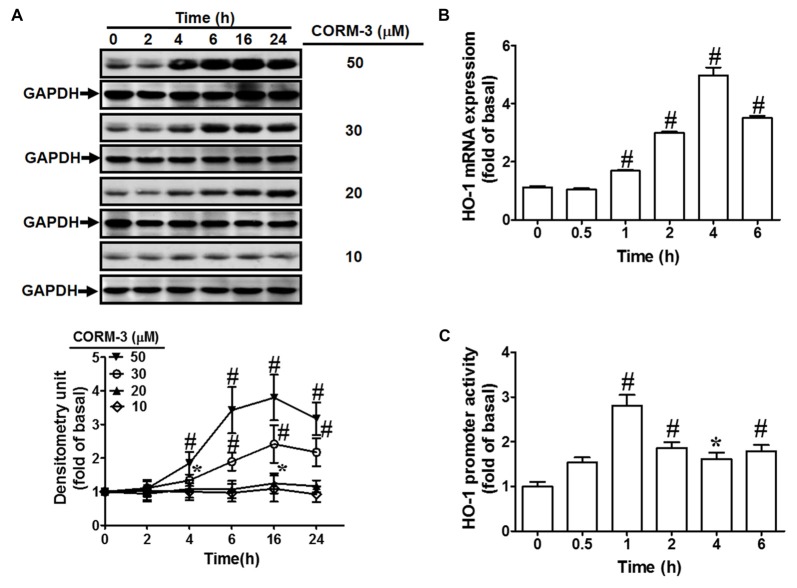
CO-releasing molecule-3 (CORM-3) induces heme oxygenase-1 (HO-1) expression.** (A)** Rat brain astrocytes (RBA)-1 cells were treated with various concentrations of CORM-3 for the indicated time intervals. The levels of HO-1 and GAPDH (as an internal control) protein expression were determined by Western blot. **(B)** Cells were treated with 30 μM CORM-3 for the indicated time intervals. The HO-1 mRNA levels were determined by real-time PCR. **(C)** Cells were co-transfected with HO-1 promoter and β-galactosidase plasmids, and then incubated with 30 μM CORM-3 for the indicated time intervals. HO-1 promoter luciferase activity was determined in the cell lysates. Data were expressed as mean ± standard errors of the mean (SEM) of three independent experiments (*n* = 3). *p < 0.05; ^#^*p* < 0.01, as compared with the cells exposed to vehicle alone.

To investigate the effects of CORM-3 on HO-1 protein and mRNA expression, RBA-1 cells were treated with various concentrations of CORM-3 (10, 20, 30, or 50 μM) for the indicated durations (0, 2, 4, 6, 16, or 24 h). Western blotting indicated that CORM-3 concentration- and time-dependently induced HO-1 protein in RBA-1 cells (Figure 1A). Significant increases occurred within 4 h of treatment and the maximal induction was observed at 16 h. Increasing concentrations of CORM-3 also produced greater HO-1 protein upregulation. These findings demonstrate that CORM-3-induced HO-1 expression is dependent on the concentration of CORM-3, as well as on treatment duration.

Next, to investigate whether CORM-3 activates HO-1 transcription, RT-PCR was performed to assess HO-1 mRNA expression. We found that CORM-3 time-dependently induced HO-1 mRNA expression and promoter activity (Figures 1B,C). HO-1 mRNA induction occurred within 1 h and reached its maximal response within 4 h of CORM-3 treatment. An HO-1 promoter containing the plasmid was used to confirm the effects of CORM-3 on HO-1 transcription. As shown in Figure 1C, CORM-3-stimulated HO-1 promoter-driven luciferase activity was also time-dependent, and the maximal response occurred within 1 h in RBA-1 cells.

### CORM-3 Induces HO-1 Expression Via Transcription and Translation Levels

**Figure 2:**
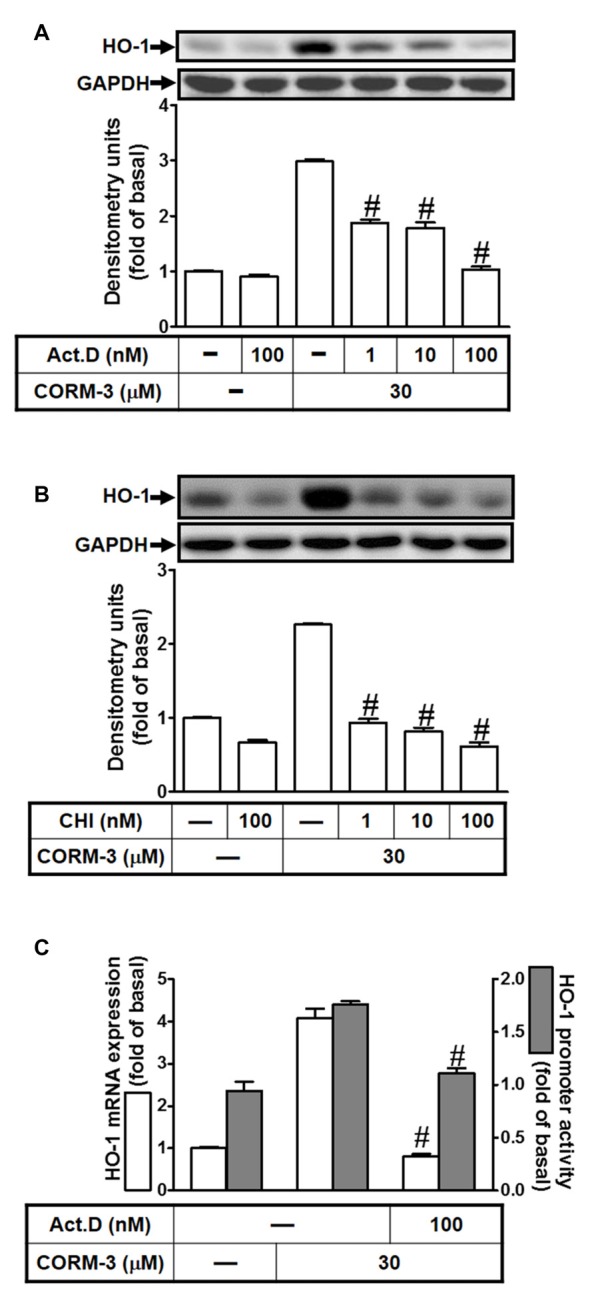
CORM-3 induces HO-1 expression via transcription and translation. RBA-1 cells were pretreated with various concentrations of either **(A)** actinomycin D (ActD) or **(B)** cycloheximide (CHI) for 1 h and then incubated with 30 μM CORM-3 for 6 h. The levels of HO-1 and GAPDH (as an internal control) protein expression were determined by Western blot. **(C)** RBA-1 cells were pretreated with 100 nM ActD for 1 h and then incubated with 30 μM CORM-3 for 4 h. The levels of HO-1 mRNA were determined by real-time PCR (open bars). Cells were transiently transfected with HO-1 report gene together a β-galactosidase plasmid, pretreated with 100 nM ActD for 1 h, and then incubated with 30 μM CORM-3 for 1 h. Promoter activity was determined in the cell lysates (shaded bars). Data are expressed as the mean ± SEM of three independent experiments (*n* = 3). ^#^*p* < 0.01, as compared with the cells incubated with CORM-3 alone.

To investigate whether CORM-3 induces HO-1 expression during transcription or translation, RBA-1 cells were pretreated with a transcription inhibitor (ActD; 1, 10, or 100 nM) or a translation inhibitor (CHI; 1, 10, or 100 nM) for 1 h, followed by 30 μM CORM-3 for 6 h. As shown in Figures 2A,B, CORM-3-induced HO-1 protein expression was concentration-dependently attenuated by pretreatment with either ActD or CHI. Furthermore, CORM-3-induced HO-1 transcription, as indicated by RT-PCR, and promoter activity, as indicated by the promoter luciferase activity assay, were also inhibited by ActD (Figure 2C). These results indicate that CORM-3-regulated HO-1 induction is mediated by transcription and translation processes in RBA-1 cells.

### CORM-3-Induced HO-1 Expression Is Mediated Via c-Src and Pyk2

**Figure 3:**
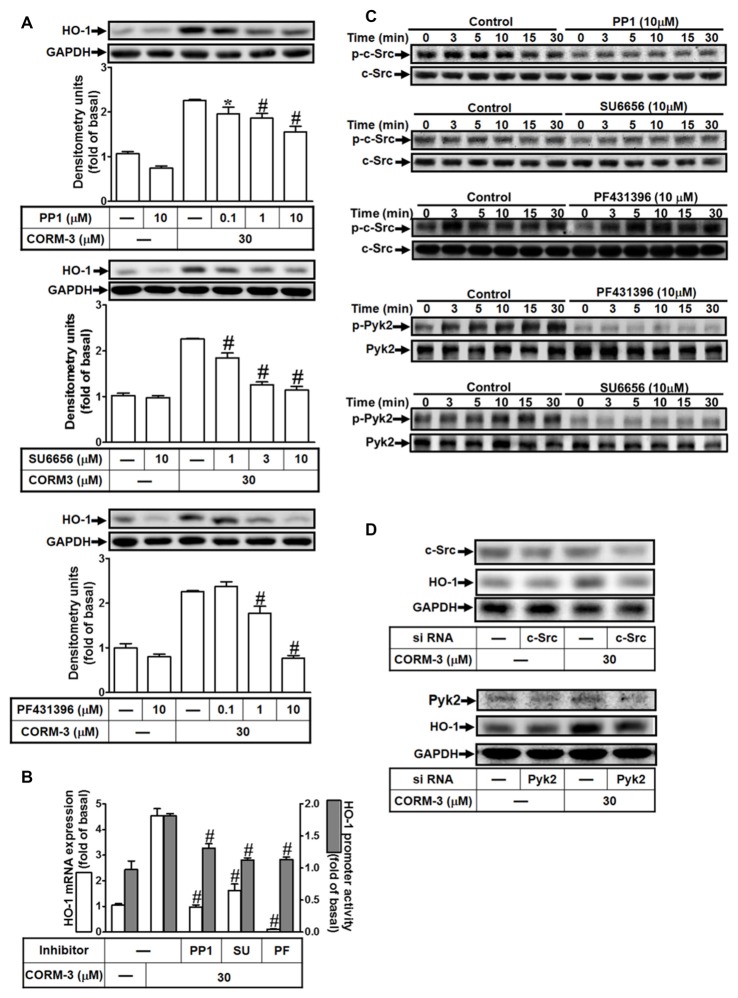
CORM-3 induces HO-1 expression via c-Src and Pyk2. **(A)** RBA-1 cells were pretreated with various concentrations of PP1, SU6656 or PF431396 for 1 h, and then incubated with 30 μM CORM-3 for 6 h. The levels of HO-1 and GAPDH (as an internal control) protein expression were determined by Western blot. **(B)** RBA-1 cells were pretreated with 10 μM PP1, 10 μM SU6656, or 10 μM PF431396 for 1 h and then incubated with 30 μM CORM-3 for 4 h. The levels of HO-1 mRNA were determined by real-time PCR (open bars). Cells were transiently transfected with HO-1 report gene together a β-galactosidase plasmid, pretreated with 10 μM PP1, 10 μM SU6656, or 10 μM PF431396 for 1 h, and then incubated with 30 μM CORM-3 for 1 h. Promoter activity was determined in the cell lysates (shaded bars). **(C)** RBA-1 cells were pretreated without or with 10 μM PP1, 10 μM SU6656, or 10 μM PF431396 for 1 h and then incubated with 30 μM CORM-3 for the indicated time intervals. The cell lysates were subjected to western blot using an anti-phospho-c-Src, anti-phospho-Pyk2, anti-c-Src, or anti-Pyk2 antibody. **(D)** Cells were transfected with c-Src siRNA or Pyk2 siRNA and then incubated with 30 μM CORM-3 for 6 h. The cell lysates were subjected to western blot using an anti-HO-1, anti-c-Src, anti-Pyk2 or GAPDH (as internal control) antibody. Data were expressed as mean ± SEM of three independent experiments (*n* = 3). *p < 0.05; ^#^*p* < 0.01, as compared with the cells exposed to CORM-3 alone.

c-Src and Pyk2 mediate HO-1 expression induced by various factors (Ryter et al., 2006). To investigate whether non-receptor tyrosine kinases including Pyk2 and c-Src contribute to CORM-3-induced HO-1 expression, RBA-1 cells were pretreated with inhibitors of c-Src (PP1, 0.1, 1, or 10 μM; or SU6656, 1, 3, or 10 μM) and PYK2 (PF431396, 0.1, 1, or 10 μM) for 1 h prior to 30 μM CORM-3 for 6 h. As shown in Figure 3A, PP1, SU6656, or PF431396 significantly and concentration-dependently decreased HO-1 induction. Furthermore, CORM-3-induced HO-1 transcription was also significantly attenuated by pretreatment with PP1, SU6656, or PF431396, as indicated by both mRNA expression and promoter activities (Figure 3B). In addition, CORM-3 time-dependently stimulated both c-Src and Pyk2 phosphorylation (Figure 3C), and these effects were attenuated by PP1 and SU6656. However, PF431396 inhibited only Pyk2 phosphorylation, and had no effect on c-Src, suggesting that Pyk2 is a downstream component of a c-Src-dependent cascade. To confirm the roles of c-Src and Pyk2 in CORM-3-induced HO-1 expression, c-Src or Pyk2 siRNA was transfected into RBA-1 cells and the effects of CORM-3 treatment on HO-1 expression were evaluated. As shown in Figure 3D, c-Src or Pyk2 siRNA transfection reduced total c-Src and Pyk2 protein, respectively and significantly reduced CORM-3-induced HO-1 expression. These results indicate that CORM-3-induced c-Src and Pyk2 activities enhance HO-1 expression in RBA-1 cells.

### CORM-3-Induced HO-1 Protein Expression Is Mediated by PKC(α)

**Figure 4:**
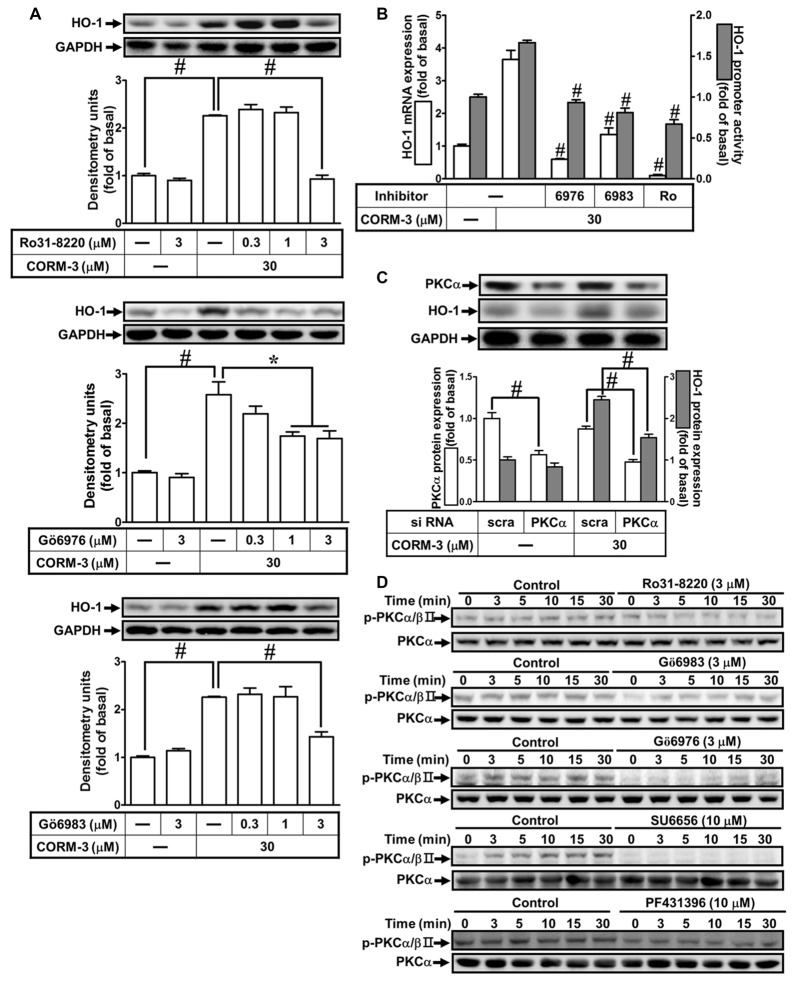
CORM-3-induced HO-1 protein expression is mediated via protein kinase C (PKCα). **(A)** RBA-1 cells were pretreated with various concentrations of Ro31-8220, Gö6976, or Gö6983 for 1 h, and then incubated with 30 μM CORM-3 for 6 h. The levels of HO-1 and GAPDH (as an internal control) protein expression were determined by Western blot. **(B)** RBA-1 cells were pretreated with 3 μM Gö6976, 3 μM Gö6983, or 3 μM Ro31-8220 for 1 h and then incubated with 30 μM CORM-3 for 4 h. The levels of HO-1 mRNA were determined by real-time PCR (Open bars). Cells were transiently transfected with HO-1 report gene together a β-galactosidase plasmid, pretreated with 3 μM Gö6976, 3 μM Gö6983, or 3 μM Ro31-8220 for 1 h, and then incubated with 30 μM CORM-3 for 1 h. Promoter activity was determined in the cell lysates (Gray bars). **(C)** RBA-1 cells were transfected with PKCα siRNA and then incubated with 30 μM CORM-3 for 6 h. The cell lysates were subjected to western blot using an anti-HO-1, anti-PKCα, or anti-GAPDH (as internal control). The densitometric quantifications are indicated on the panels. **(D)** RBA-1 cells were pretreated with 3 μM Ro31-8220, 3 μM Gö6983, 3 μM Gö6976, 10 μM SU6656, or 10 μM PF431396 for 1 h, and stimulated with 30 μM CORM-3 for the indicated time intervals. Phosphorylation of PKCα/βII was determined by Western blot using an anti-phospho-PKCα/βII or anti-PKCα antibody. Data are expressed as the mean ± SEM of three independent experiments (*n* = 3). *p < 0.05; ^#^*p* < 0.01, as compared with the cells exposed to CORM-3 alone.

Previous reports indicate that PKCs are involved in HO-1 induction (Rojo et al., 2006; Zhang and Forman, 2008; Lee et al., 2011). To investigate the roles of PKCs in CORM-3-induced HO-1 expression, RBA-1 cells were pretreated with inhibitors of PKC isoforms, as follows: PKCα/βII (Gö6976, 0.3, 1, or 3 μM) or (Gö6983, 0.3, 1, or 3 μM) and pan-PKCs (Ro31-8220, 0.1, 1, or 3 μM), for 1 h prior to 30 μM CORM-3 for 6 h. We found that Ro31-8220, Gö6976 and Gö6983 concentration-dependently suppressed HO-1 induction by CORM-3 (Figure 4A). Moreover, findings from RT-PCR and the luciferase assay also indicated that CORM-3-induced HO-1 transcription and mRNA accumulation, and that promoter activity were significantly reduced by these PKC inhibitors (Figure 4B). Next, we focused on PKCα regulation of HO-1 expression. To verify the role of PKCα in regulating HO-1 induction in RBA-1 cells treated with CORM-3, siRNA transfection was performed. As shown in Figure 4C, CORM-3-induced HO-1 expression is significantly suppressed by PKCα knockdown. Therefore, these data support that CORM-3-induced HO-1 expression is mediated by PKCα activity in RBA-1 cells. Furthermore, CORM-3 time-dependently induced phosphorylated PKCα/βII, and this effect was attenuated by pretreatment with the PKC inhibitors (Figure 4D). Moreover, we found that pretreatment with either SU6656 or PF431396 attenuated CORM-3-induced PKCα/βII phosphorylation (Figure 4D). These results demonstrate that CORM-3 stimulates c-Src/Pyk2/PKCα/βII phosphorylation and contributes to HO-1 expression in RBA-1 cells.

### CORM-3-Induced HO-1 Protein and mRNA Expression Are Mediated by p42/p44 MAPK

**Figure 5:**
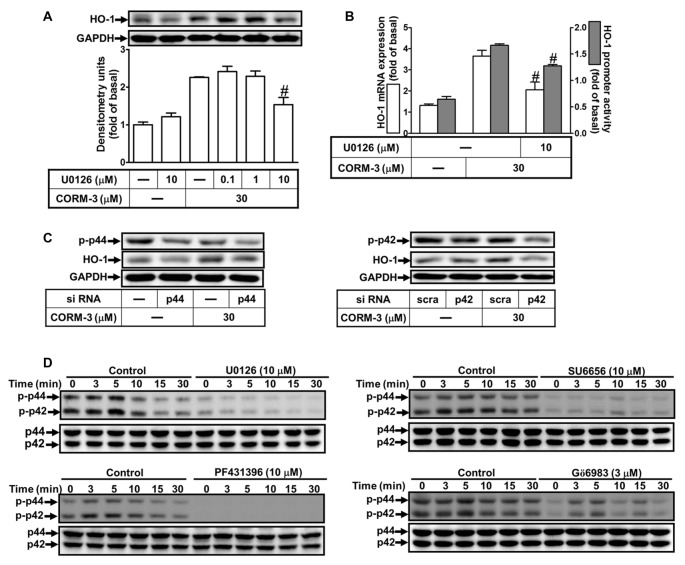
CORM-3-induced HO-1 expression is mediated through p42/p44 mitogen-activated protein kinase (MAPK).** (A)** RBA-1 cells were pretreated with various concentrations of U0126 for 1 h, and then incubated with 30 μM CORM-3 for 6 h. The levels of HO-1 and GAPDH (as an internal control) protein expression were determined by Western blot. **(B)** RBA-1 cells were pretreated with 10 μM U0126 for 1 h and then incubated with 30 μM CORM-3 for 4 h. The levels of HO-1 mRNA were determined by real-time PCR (Open bars). Cells were transiently transfected with HO-1 report gene together a β-galactosidase plasmid, pretreated with U0126 (10 μM) for 1 h, and then incubated with CORM-3 for 1 h. Promoter activity was determined in the cell lysates (Gray bars). **(C)** RBA-1 cells were transfected with p44 or p42 siRNA and then incubated with 30 μM CORM-3 for 6 h. The levels of total p42, p44 and HO-1 were determined by Western blot. **(D)** RBA-1 cells were pretreated with 10 μM U0126, 10 μM SU6656, 10 μM PF431396, or 3 μM Gö6983, for 1 h and then incubated with 30 μM CORM-3 for the indicated time intervals. Phosphorylation of p42/p44 MAPK was determined by Western blot using an anti-phospho-p42/p44 MAPK or anti-p42/p44 MAPK antibody. Data are expressed as the mean ± SEM of three independent experiments (*n* = 3). ^#^*p* < 0.05, as compared with the cells exposed to CORM-3 alone.

To determine whether CORM-3 activates p42/p44 MAPK to induce HO-1 expression, we applied a MEK1/2 inhibitor (U0126). RBA-1 cells were pretreated with U0126 (0.1, 1, or 10 μM) for 1 h prior to 30 μM CORM-3 for 6 h. We found that U0126 concentration-dependently blocked CORM-3-induced HO-1 protein expression (Figure 5A). Moreover, RT-PCR and the promoter luciferase activity assay indicated that CORM-3-induced HO-1 transcription and promoter activity were also significantly attenuated by U0126 (Figure 5B). In addition, down-regulation of p42 and p44 protein by p42 or p44 siRNA transfection suppressed CORM-3-induced HO-1 expression (Figure 5C); CORM-3 also time-dependently promoted phosphorylation of p42/p44 MAPK, which was inhibited by pretreatment with U0126 (Figure 5D). Furthermore, CORM-3-stimulated phosphorylation of p42/p44 MAPK was also attenuated by SU6656, PF431396 and Gö6983, supporting that p42/p44 MAPK is a downstream component of the c-Src/Pyk2/PKCα pathway. These results indicate that CORM-3 activates a c-Src/Pyk2/PKCα/p42/p44 MAPK pathway to induce HO-1 expression in RBA-1 cells.

### Contribution of AP-1 to CORM-3-Induced HO-1 Expression

**Figure 6:**
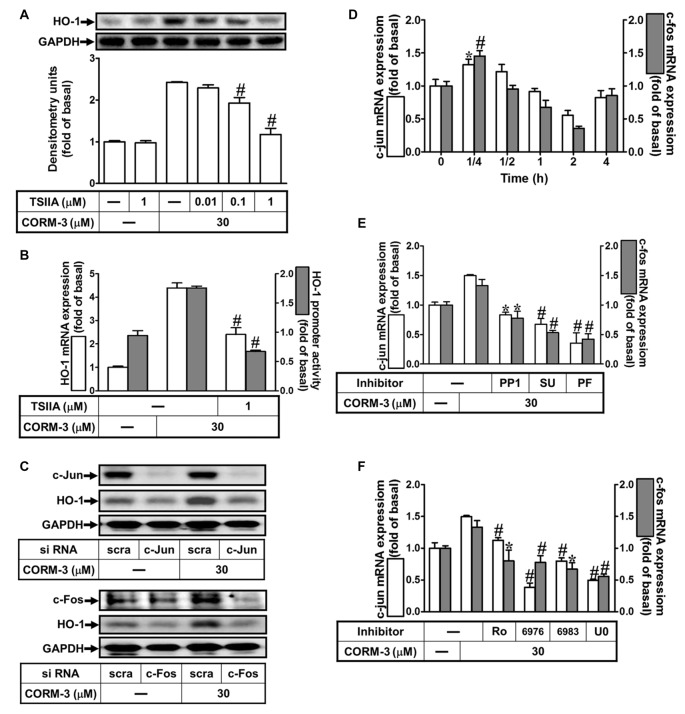
CORM-3-induced HO-1 expression is mediated through activator protein (AP)-1. **(A)** RBA-1 cells were pretreated with various concentrations of Tanshinone IIA (TSIIA) for 1 h, and then stimulated with 30 μM CORM-3 for 6 h. The levels of HO-1 and GAPDH (as an internal control) protein expression were determined by Western blot. **(B)** RBA-1 cells were pretreated with TSIIA (1 μM) for 1 h and then incubated with 30 μM CORM-3 for 4 h. The levels of HO-1 mRNA were determined by real-time PCR (Open bars). Cells were transiently transfected with HO-1 report gene together a β-galactosidase plasmid, pretreated with TSIIA (1 μM) for 1 h, and then incubated with CORM-3 for 1 h. Promoter activity was determined in the cell lysates (Gray bars). **(C)** RBA-1 cells were transfected with c-Fos or c-Jun siRNA and then incubated with 30 μM CORM-3 for 6 h. The levels of total protein c-Fos, c-Jun and HO-1 were determined by Western blot. **(D)** RBA-1 cells were incubated with 30 μM CORM-3 for the indicated time intervals. **(E,F)** Cells were pretreated without or with **(E)** PP1 (10 μM), SU6656 (10 μM), or PF431396 (10 μM) and **(F)** (3 μM) Ro-318220, (3 μM) Gö6976, (3 μM) Gö6983, or (10 μM) U0126 for 1 h and then incubated with (30 μM) CORM-3 for 15 min. **(D–F)** The levels of c-jun (Open bars) and c-fos (Gray bars) mRNA were determined by real-time PCR. Data are expressed as the mean ± SEM of three independent experiments (*n* = 3). *p < 0.05; ^#^*p* < 0.01, as compared with the cells exposed to CORM-3 alone.

Several lines of evidence indicate that activation of transcription factors such as AP-1 induces varied gene expression (Wright et al., 2009; Yang et al., 2012, 2017). To study the role of AP-1 in HO-1 induction by CORM-3, an AP-1 inhibitor (TSIIA, 0.01, 0.1, or 1 μM) was used. Pretreatment with TSIIA significantly attenuated HO-1 expression induced by CORM-3 (Figure 6A). Moreover, CORM-3-induced HO-1 transcription was also significantly inhibited by pretreatment with TSIIA, as revealed by RT-PCR and the luciferase assay (Figure 6B). In addition, knockdown of either c-Jun or c-Fos expression by siRNA transfection also abrogated HO-1 induction in CORM-3-treated RBA-1 cells (Figure 6C). To further examine whether CORM-3 affects c-Fos and c-Jun transcription, RBA-1 cells were incubated with CORM-3 and c-Fos and c-Jun mRNA expressions were analyzed using RT-PCR. As shown in Figure 6D, both c-Fos and c-Jun mRNA expressions were time-dependently upregulated by CORM-3, with maximal increases within 15 min. In addition, c-Fos and c-Jun inductions were attenuated by upstream inhibitors, including PP1, SU66656, PF431396, Ro31-8220, Gö6976, Gö6983 and U0126 (Figures 6E,F). These results suggest that HO-1 transcription is mediated by c-Src/Pyk2/PKCα/ERK1/2-dependent activation of the c-Fos and c-Jun AP-1 subunits, in CORM-3 treated RBA-1 cells.

### Upregulation of HO-1 by CORM-3 Reduces IL-1β-Induced Cell Migration

**Figure 7:**
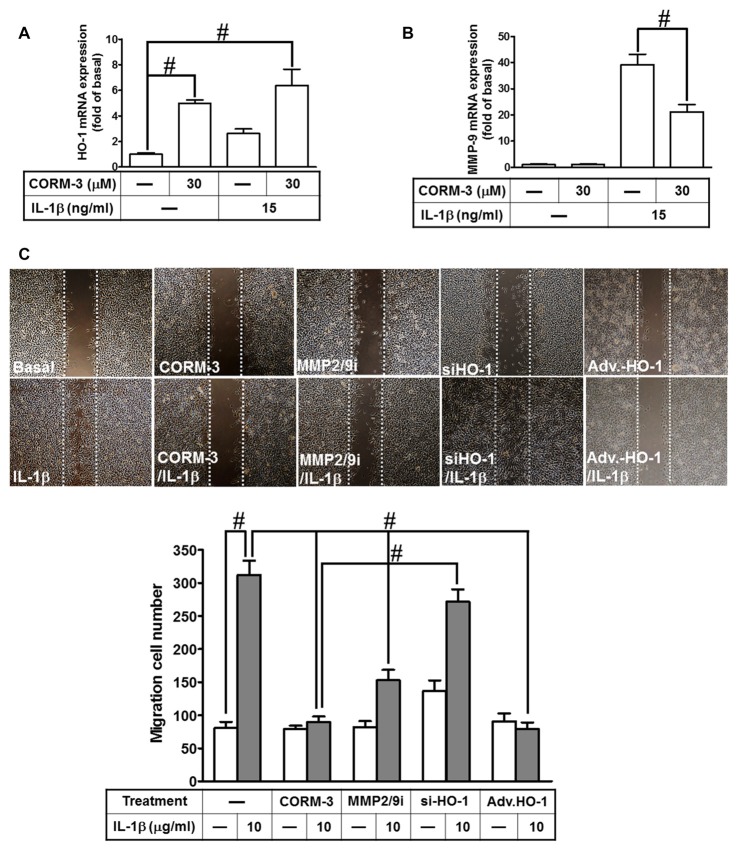
CORM-3-induced HO-1 expression reduces the interleukin (IL)-1β-induced matrix metalloproteinase-9 (MMP-9) expression and cell migration. **(A,B)** RBA-1 cells were incubated with pretreated with (30 μM) CORM-3 for 4 h, and then incubated with IL-1β (10 μg/ml) for 6 h. The levels of HO-1 and MMP-9 mRNA were determined by real-time PCR. **(C)** RBA-1 cells were pretreated with (10 μM) MMP2/9i for 1 h or transfected HO-1 siRNA followed by (30 μM) CORM-3, and infected with adenovirus-introduced HO-1, and then incubated with IL-1β (10 μg/ml) for 48 h in the presence of (1 μM) hydroxyurea. Phase contrast images of cell migration were taken at 48 h. Data are expressed as the mean ± SEM of three independent experiments (*n* = 3). ^#^*p* < 0.05, as compared with the cells exposed to **(A)** vehicle alone or **(B)** CORM-3 alone.

Our previous study indicates that MMP-9 expression promotes pathological processes in brain injury, inflammation and neurodegenerative diseases (Hsieh and Yang, 2013). Moreover, several pro-inflammatory cytokines, such as IL-1β, enhance expression of inflammatory proteins, which are implicated in brain inflammatory disorders. Our previous studies have demonstrated that IL-1β enhances cell migration resulting from MMP-9 upregulation in RBA-1 cells (Yang et al., 2015a). In addition, stimulation of the HO-1/CO system by CORM-2 reduces microglial migration and phagocytic activity induced by LPS (Scheiblich and Bicker, 2015). Therefore, we evaluated the effect of HO-1 mRNA expression induced by CORM-3 on IL-1β-stimulated cell migration. We found that HO-1 mRNA expression significantly increased with CORM-3 pretreatment, slightly increased with IL-1β and that even greater HO-1 mRNA expression occurred with the combination of CORM-3 and IL-1β (Figure 7A). Furthermore, IL-1β significantly enhanced MMP-9 transcription, which was inhibited by pretreatment with CORM-3 (Figure 7B). To elucidate the mechanisms of HO-1 regulation during the recruitment of RBA-1 cells by inflammatory cytokines, we investigated whether IL-1β induced cell motility, and whether such motility could be attenuated by CORM-3. We used an *in vitro* scratch wound assay, which measures migration during closure of a wound scratched into a confluent cell monolayer (Yang et al., 2015a). The data demonstrated that IL-1β significantly induced RBA-1 migration, which was inhibited by pretreatment with CORM-3. As predicted, overexpression of adenovirus-introduced HO-1 also inhibited IL-1β-induced cell migration; in contrast, knockdown of HO-1 expression by HO-1 siRNA or pretreatment with MMP2/9 inhibitor restored the IL-1β-mediated responses (Figure 7C). The results suggest that upregulation of HO-1 by CORM-3 attenuates IL-1β-induced cell migration by inhibiting MMP-9 activity in RBA-1 cells.

## Discussion

Upregulation of HO-1 enzymatic activity attenuates inflammatory responses and is therefore a potential target for treatment of inflammatory diseases (Willis et al., 1996). CO is a metabolite of HO-catalyzed heme degradation, and this reaction is the primary cellular source of endogenous CO. This reaction is the primary cellular source to generate endogenous CO, which is excluded the role of toxic molecule to be a widespread signaling molecule in regulation of cellular function and communication (Abraham and Kappas, 2008). CORM-3, a water soluble CORM, is an effective carrier to deliver CO and avoid toxicity in physiological conditions. In addition, several animal studies have found that CORMs have beneficial effects in the heart and lungs (Clark et al., 2003; Pak et al., 2016). However, the specific mechanisms of the signaling molecules involved in HO-1 expression induced by CORM-3 have been not completely elucidated in RBA-1 cells. In the present study, we found that CORM-3 activated the c-Src/Pyk2/PKCα/ERK1/2-dependent signal transduction pathway and c-Fos and c-Jun (AP-1) transcription, which resulted in HO-1 induction in RBA-1 cells. Furthermore, upregulation of HO-1 attenuated IL-1β-induced cell migration by inhibiting MMP-9 expression and activity in RBA-1 cells.

Our previous studies and research conducted by others indicate that activation of c-Src- and Pyk2-dependent pathways regulates HO-1 induction in human, rat and bovine cells (Choi and Alam, 1996; Han et al., 2009; Chi et al., 2015; Yang et al., 2015b). In the present study, we found that CORM-3-induced HO-1 expression was mediated by activation of c-Src and Pyk2, which was confirmed using pharmacological inhibitors such as PP1, SU6656 and PF431396, as well as by transfection with the respective siRNAs. The present results are consistent with our previous studies indicating that CORM-2-induced HO-1 expression is mediated by c-Src or Pyk2 in various cell types (Chi et al., 2015; Chien et al., 2015; Yang et al., 2015b). The relationship between c-Src and Pyk2 in CORM-3-mediated responses was elucidated using the respective inhibitors and siRNAs. The inhibitors of c-Src (PP1 and SU6656) attenuated CORM-3-stimulated phosphorylation of both c-Src and Pyk2, whereas the inhibitor of Pyk2 (PF431396) blocked Pyk2 phosphorylation only. These findings suggest that c-Src is an upstream component of Pyk2 in the CORM-3-mediated response. Therefore, our results support that CORM-3-induced HO-1 expression is mediated by a c-Src/Pyk2-dependent pathway in RBA-1 cells. In addition, upregulation of HO-1 expression by acrolein and fisetin is mediated by PKCδ activation (Zhang and Forman, 2008; Lee et al., 2011), and NGF induces HO-1 by activating PKCζ in PC12 cells (Rojo et al., 2006). These studies indicate that multiple PKC isoforms contribute to HO-1 regulation. These findings may be due to the different cell types and various HO-1 inducers applied across studies. In the present study, we also demonstrated that PKCα is activated downstream of c-Src/Pyk2 using the inhibitors PP1, SU6656, PF431396, or Gö6976 with RBA-1 cells.

Loss of control or abnormal MAPK functions are implicated in inflammatory responses and tissue injury, which results in dysregulated expression of pro- or anti- inflammatory mediators (Cheng et al., 2010; Alam and Gorska, 2011; Lee and Yang, 2013). In the present study, we demonstrated that activation of ERK1/2 contributes to CORM-3-induced HO-1 expression, which is attenuated by a selective MEK1/2 inhibitor (U0126) or gene-silencing by p42 or p44 siRNA transfection. Our results are consistent with previous reports that ERK1/2 activities are involved in HO-1 expression induced by hypoxia in rat pulmonary aortic endothelial cells (Ryter et al., 2002), as well as by hydrogen sulfide (Oh et al., 2006) and bisdemethoxycurcumin (Kim et al., 2010) in RAW264.7 macrophages, and resveratrol in PC12 cells (Chen et al., 2005). Moreover, the results obtained from the inhibitor experiments indicate that c-Src (SU6656), Pyk2 (PF431396) and PKCα (Gö6983), significantly attenuate CORM-3-stimulated phosphorylation of ERK1/2, suggesting that a c-Src/Pyk2-dependent PKCα cascade is required for CORM-3-stimulated ERK1/2 phosphorylation in RBA-1 cells.

Increased oxidative stress during cell injury or microbial infection produces macromolecule damage to the cell membrane, chromosome breakdown, or protein dysfunction. In contrast, ROS generation also modulates oxidative-responsible gene expression via signal transduction, thereby mediating the functions of transcription factors; ROS-dependent signal results in the transcription factors interacting with response elements on the HO-1 promoter to regulate gene transcription (Rochette et al., 2013). However, the upstream or downstream signaling components that mediate the HO-1 regulating CORM-3 responses have not been completely elucidated in RBA-1 cells. Heterodimers of AP-1 are important transcription factors for regulating downstream gene expression and mediating intracellular redox states (Kiemer et al., 2003). In addition, AP-1 components are activated by oxidative stress-associated protein kinases, and bind to response elements of promoters on inflammatory genes (Sen and Packer, 1996). In the present study, the involvement of AP-1 in these responses was supported by the finding that CORM-3-induced HO-1 was inhibited by an AP-1 inhibitor (TSIIA). In addition to the inhibitor treatment, the regulatory functions of c-Fos, c-Jun and AP-1 were also confirmed with gene-silencing via siRNA transfection. These consistent results indicate that reductions of either c-Fos or c-Jun significantly attenuate CORM-3-induced HO-1 expression. We also demonstrated that CORM-3-stimulated both c-Fos and c-Jun mRNA expressions, which were reduced by inhibitors of upstream kinases such as PP1, SU6656, PF431396, Ro-31-8220, Gö6976, Gö6983, or U0126, indicating that CORM-3 upregulates c-Fos and c-Jun mRNA via a c-Src/Pyk2/PKCα/p42/p44 MAPK pathway. The present results are consistent with reports indicating that upregulation of HO-1 transcription is mediated by either induction of c-Jun and c-Fos, or by AP-1 activity in various cell types (Kiemer et al., 2003; Cheng et al., 2006; Hsieh et al., 2010). Furthermore, upregulation of HO-1 protected against IL-1β-induced cell migration, via inhibition of MMP-9 activity in RBA-1 cells, which is consistent with evidence for endogenous endothelial progenitor cell recruitment in myocardium of hyperglycemic streptozotocin-treated rats (Filippo et al., 2012).

Species differences in HO-1 expression induced by CORMs and various other stimuli have not been determined. For example, heat stress upregulates HO-1 expression in most species, although this effect does not occur in human cells (Okinaga et al., 1996). In addition, hypoxia upregulates HO-1 expression in most species but suppresses it in human cells (Kitamuro et al., 2003). In the present study, CORM-3 induced HO-1 expression and exerted anti-inflammatory effects in RBA-1 cells. Our previous studies also indicate that CORM-2 induces HO-1 expression in human tracheal smooth muscle cells (Yang et al., 2015b), human cardiomyocytes (Chien et al., 2015), and RBA-1 cells (Chi et al., 2015). The differences in HO-1 induction patterns may result from species differences and varied experimental conditions. CORM-3 has anti-inflammatory effects, which are mediated by suppressing the interaction with MAPKs and transcription factors, thereby increasing HO-1 expression. Furthermore, CORM-3 has a wide range of functions that produce anti-inflammatory and anti-oxidant effects to reduce nitrite and tumor necrosis factor-α release from BV-2 microglia cells (Bani-Hani et al., 2006) and murine macrophages (Sawle et al., 2005). In contrast, several reports also support that nitric oxide donors contribute to HO-1 expression in human cells (Hara et al., 1999; Kitamuro et al., 2003). HO-1 induction is a protective mechanism against oxidative stress. The specific mechanisms of CORM-3 signaling in protection against neuroinflammation should be addressed by future investigations.

**Figure 8:**
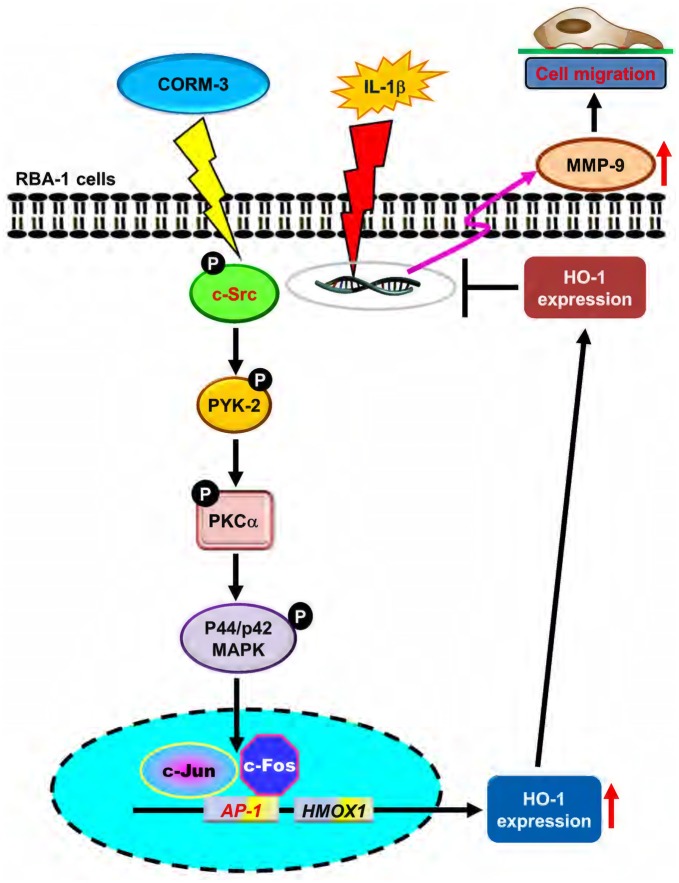
Schematic representation of signaling pathways involved in CORM-3-induced HO-1 expression and protected against IL-1β-induced cell migration in RBA-1 cells. CORM-3 activated c-Src/Pyk2/PKCα/p42/p44 MAPK/AP-1 pathway to induce HO-1 expression which suppressed the IL-1β-induced MMP-9 mRNA expression and cell migration.

The schematic signaling pathways elucidated in the present study are illustrated in Figure 8. In summary, we found that CORM-3 initiated a c-Src/Pyk2/PKCα/p42/p44 MAPK-dependent AP-1 cascade, and resulted in HO-1 expression in RBA-1 cells. Upregulation of HO-1 attenuates IL-1β-induced cell migration via inhibition of MMP-9 expression in RBA-1 cells and is therefore a potential strategy for treatment of inflammatory brain diseases.

## Author Contributions

C-CL, C-CY, L-DH, S-YC and C-MY substantially contributed to the conception or design of the work, the acquisition, analysis and interpretation of data for the work, finally approved the version to be published and agreed to be accountable for all aspects of the work in ensuring that questions related to the accuracy or integrity of any part of the work are appropriately investigated and resolved. C-CL, C-CY and C-MY drafted the work and revised it critically for important intellectual content.

## Conflict of Interest Statement

The authors declare that the research was conducted in the absence of any commercial or financial relationships that could be construed as a potential conflict of interest.
